# Overcoming Biological Barriers: Importance of Membrane Transporters in Homeostasis, Disease and Disease Treatment

**DOI:** 10.3390/ijms24087212

**Published:** 2023-04-13

**Authors:** Giuliano Ciarimboli

**Affiliations:** Experimental Nephrology, Department of Internal Medicine and Nephrology, Medical Clinic D, University Hospital Münster, 48149 Münster, Germany; gciari@uni-muenster.de

## 1. Reviews

This editorial summarizes the 22 scientific papers published in the Special Issue “Overcoming Biological Barriers: Importance of Membrane Transporters in Homeostasis, Disease, and Disease Treatment” of the International Journal of Molecular Sciences. In this Special Issue, the readers will find integrative information in the field of transporters research. Transporters play an important role for signal molecules, nutrients, metabolites, xenobiotics, and drugs to overcome the barrier posed by biological membranes. Therefore, transporters help in maintaining homeostasis and in the handling of drugs. Changes in the function of transporters can impair homeostasis, cause disease, or modify the efficacy of disease treatment with drugs. This Special Issue aims to collect the newest information on transporters, with a special focus on their function and regulation, their pathological roles, and their importance for drug effects and unwanted side effects. In addition to 4 reviews, readers will find 18 original research works focusing on specific aspects of transporter physiology and pharmacology.

The review “Inborn Errors of Nucleoside Transporter (NT)-Encoding Genes (*SLC28* and *SLC29*)” by Pastor-Anglada et al. [1] summarizes the genetic alterations impacting nucleoside and nucleobase transporters, focusing on *Solute Carrier* (*SLC*) *29A1*, *SLC29A3*, and *SLC28A1*, and underlines the relationships between transporter function and pathology.

In mammalian cells, 14 different transporters for glucose and fructose exist (glucose transporters, GLUTs), suggesting potential additional roles of these transporters in cellular physiology and pathology. In the review by Ismail and Tanasova [2], the roles of GLUTs in disease and disease treatment are well summarized. 

Nonalcoholic fatty liver disease (NAFLD) is found in a high number of people and seems to be associated with the development of hepatocellular carcinoma (HCC). Since the transmembrane 4 L six family member 5 (TM4SF5) is a possible biomarker for NAFLD, the review by Kim et al. [3] summarizes the relationships between TM4SF5 and other nutrient transporters in hepatocytes, which can be important in the development of chronic liver diseases. 

Finally, the review by Xiu et al. [4] focuses on the role of organic cation transporters for pharmacokinetics, pharmacodynamics, and drug–drug interactions of tyrosine kinase inhibitors, which are drugs with high importance in the treatment of cancer.

## 2. Original Research Works

In the paper “Evolution of the Membrane Transport Protein Domain”, Dabravolski and Isayenkov [5] used different bioinformatic tools to investigate the evolution of membrane transport proteins, analyze their domain organization and loop topology, and to compare the alignment of modelled 3D structures. 

Starting from the observation that nucleoside and/or nucleobase transporters are essential for the growth and proliferation of all protozoan parasites and considering that these transporters are potential targets for a drug therapy, Aldfer et al. [6] described in the manuscript “Nucleoside Transport and Nucleobase Uptake Null Mutants in *Leishmania mexicana* for the Routine Expression and Characterization of Purine and Pyrimidine Transporters” the development of a system for the expression of nucleoside transporters in *Leishmania mexicana*, where all endogenous nucleoside transporters were deleted by clustered regularly interspaced short palindromic repeats (CRISPR)/CRISPR-associated protein 9 (Cas9) (CRISPR/Cas9) technology. This system provides an ideal null background for the expression and characterization of single equilibrative nucleoside transporter (ENT) genes. Using the same null background *Leishmania mexicana* cell line, in the manuscript “Cloning and Characterization of *Trypanosoma congolense* and *T. vivax* Nucleoside Transporters Reveal the Potential of P1-Type Carriers for the Discovery of Broad-Spectrum Nucleoside-Based Therapeutics against Animal African Trypanosomiasis” Ungogo et al. [7] investigated the druggability of the main adenosine transporters (purine nucleoside transporter NT3) of *T. vivax* (TvxNT3) and *T. congolense* (TcoAT1/NT10) by 7-substituted tubercidins and other nucleoside analogs. They showed that chemotherapy for Animal African Trypanosomiasis is possible. 

In a work with prokaryotic transporters entitled “An Efficient Way to Screen Inhibitors of Energy-Coupling Factor (ECF) Transporters in a Bacterial Uptake Assay”, Bousis et al. [8] investigated inhibitors of energy-coupling factor (ECF) transporters using *Lactobacillus casei* as a model microorganism. ECF transporters facilitate the uptake of micronutrients such as B-type vitamins and cations inside the cell. The screening system with *Lactobacillus casei* allowed the rapid identification of novel inhibitors of ECF transporters and their optimization based on structure–activity relationships. The paper “Generalized Approach towards Secretion-Based Protein Production via Neutralization of Secretion-Preventing Cationic Substrate Residues” by Byun at al. [9] is another study on bacterial transporters. Bacterial ATP-binding cassette (ABC) transporters are able to secrete heterologous proteins. However, some proteins are not secretable. The authors presented a method to induce the secretion of these non-secretable proteins, which provides new hints on the secretory physiology of ABC transporters and is also important for industrial applications. 

A further paper dedicated to the establishment of a suitable system for investigating the function and interaction with drugs of a specific transporter is the one by Temesszentandrási-Ambrus et al. [10]. In the paper “A Unique In Vitro Assay to Investigate ABCB4 Transport Function”, they describe the development of a Madin-Darby Canine Kidney II (MDCKII) cell line, where the endogenous expression of permeability glycoprotein 1 (P-gp, also known as multidrug resistance protein 1 (MDR1) or ATP-binding cassette sub-family B member 1 (ABCB1)) was deleted using CRISPR/Cas9 technology to generate ABCB1 knockout (KO) MDCKII cells. After this, the multidrug resistance protein 3 (MDR3 or ATP-binding cassette sub-family B member 4 (ABCB4)) was transduced in ABCB1-KO-MDCKII cells and its activity measured using digoxin as a substrate. This system was revealed to be suitable to study the interaction of several hepatotoxic, anticancer, as well as ABCB1 interactor drugs with ABCB4-mediated digoxin transport.

Further papers of the Special Issue focused on the interaction of transporters with drugs and xenobiotics. In the manuscript “Interaction of Masitinib with Organic Cation Transporters”, Harrach et al. [11] showed that the tyrosine kinase inhibitor Masitinib is a substrate for organic cation transporters. Considering that Masitinib is a potent inhibitor of severe acute respiratory syndrome coronavirus-2 (Sars-CoV-2) cellular replication, these findings predict higher efficacy of this treatment in cells expressing these transporters. The manuscript “Investigations with Drugs and Pesticides Revealed New Species- and Substrate-Dependent Inhibition in Human and Mouse Organic Cation Transporter OCT2” by Kuehne et al. [12] compared the interaction of 13 drugs and 9 pesticides with the human and mouse organic cation transporter 2 (hOCT2 and mOct2, respectively). The OCT2 is a transporter that mediates the renal tubular secretion of cationic substances. They found species-dependent differences for 2 and substrate-dependent differences for 3 out of 22 investigated compounds, showing that species-dependent differences should be considered when extrapolating data from mice to humans. In the paper “MATE1 Deficiency Exacerbates Dofetilide-Induced Proarrhythmia”, Uddin et al. [13] found that Dofetilide, a class III antiarrhythmic drug, is a substrate of the multidrug and toxin extrusion protein 1 (MATE1). Dofetilide transport in cardiomyocytes and renal tubular cells is mediated by MATE1 and is highly sensitive to pharmacological inhibition, suggesting that these findings are important for optimizing polypharmacy regimens. Monocrotaline (MCT) is a pyrrolizidine alkaloid that is present in plants and can damage the liver of several organisms, such as rats, inducing sinusoidal obstruction syndrome (SOS). In the work “Monocrotaline Toxicity Alters the Function of Hepatocyte Membrane Transporters in Rats” by Pastor and Vilgrain [14], the authors investigated the effect of MCT administration to rats, which induces SOS, on the biliary excretion of gadobenate dimeglumine (BOPTA), a hepatobiliary substrate used in clinical imaging. The authors showed that SOS is associated with the dysfunction of liver efflux transporters.

Pharmacological intervention on transport processes can help to treat transporter-associated pathologies, how Tonum et al. showed in the manuscript “Pharmacological Effects of Panduratin A on Renal Cyst Development in In Vitro and In Vivo Models of Polycystic Kidney Disease” [15]. In this work, they demonstrated that the activation of 5’ AMP-activated protein kinase (AMPK) with Panduratin A inhibits cystic fibrosis transmembrane conductance regulator (CFTR)-mediated chloride secretion and reduces renal cyst expansion in polycystic kidney disease (PKD) in vitro and in vivo. The in vivo efficacy of Panduratin A treatment is dependent on the route of administration.

Since transporters can be the target of pharmacological disease treatment and can determine drug disposition, it is important to know whether and how pathological states can influence transporter functions. Idowu and Hagebuch addressed this point in their paper “Free Cholesterol Affects the Function and Localization of Human Na^+^/Taurocholate Cotransporting Polypeptide (NTCP) and Organic Cation Transporter 1 (OCT1)” [16]. They showed that increased cholesterol levels, which are observed in NAFLD and in nonalcoholic steatohepatitis (NASH) can impair the function of NTCP and OCT1, two important hepatic transporters, and may therefore change drug disposition in the liver. The paper “Protein Abundance of Drug Transporters in Human Hepatitis C Livers“ by Droździk et al. [17] describes the measurement of abundance of several transporters by liquid chromatography-tandem mass spectrometry (LC-MS/MS) in human hepatitis C virus (HCV)-infected liver samples. The abundances of P-gp, multiple drug-resistance-associated protein (MRP) 1 (MRP1), breast cancer resistance protein, and organic anion transporting polypeptide 1 (OATP1)-B3 (OATP1B3) protein were upregulated, whereas those of MRP2, MRP4, NTCP, OATP2B1, and OCT1 were downregulated in all HCV samples. These results are important to improve the pharmacological treatment of HCV patients. 

NTCP (encoded by *SLC10A1*) is the main hepatic uptake transporter for conjugated bile salts in humans. Through its expression on the sinusoidal hepatocyte membrane and on the apical membrane of enterocytes, NTCP plays an important role for the enterohepatic circulation of bile salts. Additionally, NTCP is involved in the transport of several different drugs and is also the receptor for cellular entry of the human hepatitis B and D viruses (HBV and HDV). In the paper “Hepatic Expression of the Na^+^-Taurocholate Cotransporting Polypeptide Is Independent from Genetic Variation”, Tremmel et al. [18] investigated interindividual patient’s hepatic SLC10A1/NTCP expression using various omics technologies and found that the variability in NTCP expression is multifactorial with the contribution of clinical factors, DNA methylation, transcriptional regulation, as well as hepatic metabolism, but not of genetic variation.

The OCT1 is an important polyspecific transporter expressed in the sinusoidal hepatocyte membrane. Because of its specificity, it can interact with many different substrates. In the work “Relationships between Inhibition, Transport and Enhanced Transport via the Organic Cation Transporter 1”, Jensen et al. [19] characterized in detail the interaction of OCT1 with many different substances. They showed that OCT1 substrates are mainly weak OCT1 inhibitors, and among those substrates with stronger inhibiting properties, the type of inhibition may be noncompetitive. Therefore, OCT1 inhibition screenings probably poorly predict OCT1 substrates. Interestingly, the OCT1 substrate sumatriptan can enhance the uptake of some other OCT1 substrates. This phenomenon has also been observed in other studies with OCTs; however, it is still not fully understood. According to the authors, this apparent uptake stimulation may be caused by a combination of different inhibition strengths of OCT1-mediated uptake and efflux. 

It is known that liver injury can lead to hepatic retinopathy. To investigate the mechanisms of hepatic retinopathy, in the paper “Bile Duct Ligation Impairs Function and Expression of Mrp1 at Rat Blood–Retinal Barrier via Bilirubin-Induced P38 MAPK Pathway Activations“, Li et al. [20] used a bile duct ligation (BDL) liver injury model to study the retinal expression and function of Mrp1, which is important to clear retinal toxins. They were able to show that retinal Mrp1 function and expression in the BDL model of liver injury are downregulated by the activation of the p38-mitogen-activated protein kinases (MAPK) caused by increased bilirubin levels. The transport activity of mouse Mrp1 was measured in vivo by means of positron emission tomography (PET) imaging with the Mrp1 tracer 6-bromo-7-[^11^C]methylpurine, as described in the paper “Use of PET Imaging to Assess the Efficacy of Thiethylperazine to Stimulate Cerebral MRP1 Transport Activity in Wild-Type and APP/PS1-21 Mice” by Wölfl-Duchek et al. [21]. They found that treatment with thiethylperazine (an antiemetic drug which was shown to stimulate MRP1 activity in vitro) had no significant effect on cerebral and pulmonary Mrp1 activity in both wild-type and APP/PS1-21 mice, a rapid β-amyloidosis mouse model.

Focusing on renal transporters, the paper “In Vitro Characterization of Renal Drug Transporter Activity in Kidney Cancer” by Caetano-Pinto et al. [22] investigated transporter expression and function in different renal cell carcinoma (RCC) cell lines and in a prototypical non-malignant renal proximal tubule epithelial cell (RPTEC) line. They found that the RCC cell lines showed differential expression and function of renal drug transporters. Transporter expression was differently regulated by methylation and EGFR inhibition. The expression and activity of kidney transporters in RPTEC cells were limited when compared to those in primary RPTEC.
